# Complete chloroplast genome of *Toona sinensis* (Meliaceae), a goluptious ‘tree vegetables’

**DOI:** 10.1080/23802359.2019.1666664

**Published:** 2019-09-17

**Authors:** Bingbing Liu, Junfeng Zhang, Yancai Shi

**Affiliations:** a Institute of Loess Plateau, Shanxi University, Taiyuan, China;; b Guangxi Key Laboratory of Plant Conservation and Restoration Ecology in Karst Terrain, Guangxi Institute of Botany, Guangxi Zhuang Autonomous Region and Chinese Academy of Sciences, Guilin, China

**Keywords:** *Toona sinensis*, chloroplast genome, phylogenetic analysis

## Abstract

*Toona sinensis*, also known as Xiangchun in Chinese, is a popular ‘tree vegetables’ and famous medicinal plant with good economic value. In our study, we sequenced the complete chloroplast (cp) genome of *T. sinensis* using the llumina sequencing platform. The cp genome of *T. sinensis* is a characteristic four-party structure with a length of 157,228 bp, which contains two 26,994 bp inverted repeats (IRs), an 85,971 bp large single copy (LSC), and a 17,269 bp small single copy (SSC). We identified a total of 126 genes, of which clouding 82 protein-coding genes, 36 tRNA genes, and eight rRNA genes. The phylogenetic analysis showed that *T. sinensis* was closely related to the congeneric *T. ciliata*.

*Toona sinensis* (A. Juss.) M. Roem. (TS), commonly named Chinese Xiangchun, is a perennial, deciduous tree belongs to the family of Meliaceae. It is widely distributed in eastern and southeastern Asia, e.g., in central and southern China (Wu et al. 2012). *Toona sinensis* is compliment as ‘tree vegetables’ due to possesses a unique and pleasant flavor and is consumed as a popular seasonal vegetable in certain parts of eastern and southeastern Asia (Hu et al. 2016). *Toona sinensis* is also a famous medicinal plant in China. Its young leaves have various biological and pharmacological functions, including anti-cancer, anti-diabetic, anti-viral, and antioxidant properties (Yang et al. 2019). Its economic importance can exemplarily be seen in the fact that >1 million people are actually working on *T. sinensis* products in China. Its planting area is >1 billion square meters and cultivating >800 billion kg of *T. sinensis* sprouts per year (Zhai and Granvogl 2019). However, there is little genomic information has been reported. Herein, we first report the complete chloroplast genome of *T. sinensis*, which might provide comprehensive information relevant to the effective conservation and management of this species.

The tender leaves of *T. sinensis* were sampled from the school garden of Shanxi University (Shanxi, China; 112°34′12″ E, 37°43′48″ N) and were used for the total genomic DNA extraction with a DNeasy Plant Mini Kit (QIAGEN, Valencia, California, USA). The voucher specimen (liu2018015) and the DNA sample (TS-2) of *T. sinensis* were deposited in the Molecular and Physiological Ecology Laboratory, Institute of Loess Plateau, Shanxi University (Taiyuan, Shanxi, China). The whole-genome sequencing was conducted with 150 bp pair-end read on the Illumina Hiseq Platform (Illumina, San Diego, CA). In total, about 400 million high-quality clean reads are obtained and used for the cp genome de novo assembly by the program NOVOPlasty (Dierckxsens et al. 2017) and direct-viewing in Geneious R11 (Biomatters Ltd., Auckland, New Zealand). Annotation was performed with the program Plann (Huang and Cronk 2015) and Sequin (http://www.ncbi.nlm.nih.gov/). Together with gene annotations, the complete cp genome sequences were submitted to GenBank and deposited under the accession number MK954108. A neighbour-joining (NJ) tree with 1000 bootstrap replicates was inferred using MEGA version 6 (Tamura et al. 2013) from alignments created by the MAFFT (Katoh and Standley 2013) using plastid genomes of 21 species.

The chloroplast genome of *T. sinensis* is a typical quadripartite structure with a length of 157,228 bp, which contains two 26,994 bp inverted repeats (IRs), and the IRs were parted by a large single copy (LSC) (85,971bp) and a small single copy (SSC) (17,269 bp). We identified a total of 126 genes, of which clouding 82 protein-coding genes, 36 tRNA genes, and eight rRNA genes. Among the annotated genes, three of them contain three introns (*trn*A-UGC, *trn*I-GAU, *rps*12), one gene (*clp*P) contains two introns, and seven genes (*trn*I-CAU, *trn*L-CAA, *trn*V-GAC, *trn*N-GUU, *trn*R-ACG, *ndh*B, and ycf2) contain one intron. The overall GC content of the plastome is 37.9% while the corresponding values of the LSC, SSC, and IR regions are 36.1%, 32.1%, and 42.7%, respectively. The phylogenetic analysis suggested that *T. sinensis* was closely relevant to the species of *T. ciliata* (Figure 1).

**Figure 1.**
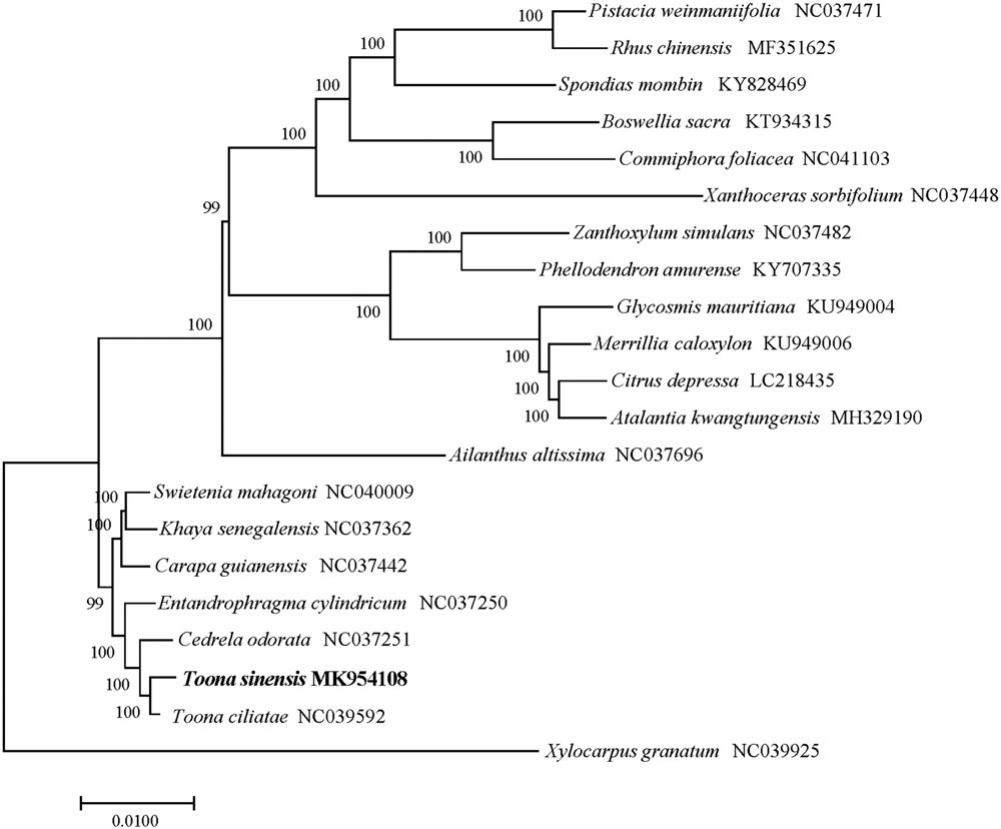
The neighbour-joining (NJ) tree based on the 21 chloroplast genomes. The bootstrap value based on 1000 replicates is shown on each node.

This absolute cp genome can be afterward used for population, phylogeny, and cp genetic project studies of *T. sinensis* and such cognition would be fundamental to the potential development value and provide some help for the large-scale breeding of *T. sinensis*.
